# Drought and Recovery: Independently Regulated Processes Highlighting the Importance of Protein Turnover Dynamics and Translational Regulation in Medicago truncatula*[Fn FN2]

**DOI:** 10.1074/mcp.M115.049205

**Published:** 2016-03-21

**Authors:** David Lyon, Maria Angeles Castillejo, Vlora Mehmeti-Tershani, Christiana Staudinger, Christoph Kleemaier, Stefanie Wienkoop

**Affiliations:** From the ‡Department of Molecular Systems Biology, University of Vienna, Vienna, Austria

## Abstract

Climate change in conjunction with population growth necessitates a systems biology approach to characterize plant drought acclimation as well as a more thorough understanding of the molecular mechanisms of stress recovery. Plants are exposed to a continuously changing environment. Extremes such as several weeks of drought are followed by rain. This requires a molecular plasticity of the plant enabling drought acclimation and the necessity of deacclimation processes for recovery and continuous growth.

During drought stress and subsequent recovery, the metabolome and proteome are regulated through a sequence of molecular processes including synthesis and degradation and molecular interaction networks are part of this regulatory process. In order to study this complex regulatory network, a comprehensive analysis is presented for the first time, investigating protein turnover and regulatory classes of proteins and metabolites during a stress recovery scenario in the model legume *Medicago truncatula*. The data give novel insights into the molecular capacity and differential processes required for acclimation and deacclimation of severe drought stressed plants.

Functional cluster and network analyses unraveled independent regulatory mechanisms for stress and recovery with different dynamic phases that during the course of recovery define the plants deacclimation from stress. The combination of relative abundance levels and turnover analysis revealed an early transition phase that seems key for recovery initiation through water resupply and is independent from renutrition. Thus, a first indication for a metabolite and protein-based load capacity was observed necessary for the recovery from drought, an important but thus far ignored possible feature toward tolerance. The data indicate that apart from the plants molecular stress response mechanisms, plasticity may be related to the nutritional status of the plant prior to stress initiation. A new perspective and possible new targets as well as metabolic mechanisms for future plant-bioengineering toward enhanced drought stress tolerance are presented.

Grain and forage legumes represent some of the most important crop species worldwide, accounting for 27% of the world's primary crop production (1). Despite the numerous agronomical and environmental advantages of legumes, their production is limited by abiotic stresses, particularly low water availability (2). Drought has a major impact on crop yield, affecting the majority of the agricultural regions around the world.

In general, drought stress negatively affects almost all aspects of plant metabolism, inducing a number of changes at the morphological, physiological, biochemical, and metabolic levels in all plant organs (3). Photosynthesis and cell growth are among the primary processes to be affected by drought (4). Deleterious effects of drought on photosynthesis will be mediated by the responsiveness of (1) the respiration system, electron transport, and ATP synthesis in the mitochondria (5), (2) gene expression and protein turnover (3), and (3) the accumulation of stress metabolites (6).

Stress avoidance mechanisms essentially aim at maintaining the initial plant water status and lowering the rate of stress imposed at the tissue or cellular level. Tolerance strategies aim at preventing damage and maintaining metabolism, once water deficit has been established. Most of these strategies involve dynamic changes in protein abundance that can be best explored through proteomics. Proteomic studies conducted with roots of several plant species subjected to drought and other abiotic stresses have been reviewed in (7).

Stress recovery strategies aim at re-adjusting to an initial metabolic state and depending on the severity of stress to overcome stress induced senescence mechanisms of remaining cells and tissues. Even though, much is known about drought stress, its complexity makes it difficult to find suitable biomarkers for successful crop-engineering or smart-breeding of increased drought-tolerant plants.

*Medicago truncatula* is an annual forage model legume with highest frequency in the arid and semi-arid areas of the Mediterranean; given its drought-adaptive nature, it is an ideal candidate to study the molecular and biochemical mechanisms facilitating drought tolerance in plants (8). Many studies on drought stress effects have recently been conducted on *Medicago spp.* (9–12). However, only few studies have been conducted on plant responses to drought stress recovery (rewatering) and their underlying mechanisms (13–16). An integrative transcript and metabolite analysis during progressive drought and rewatering was recently published (17). This technical combination highlighted the important regulatory impact on metabolic pathways involvement in *M. truncatula* drought tolerance with the common stress metabolites myo-inositol and proline. Integrative studies on the *M. truncatula* root nodule metabolome and proteome during drought stress and recovery revealed some general protein breakdown connected to the severity of stress and a subsequent increase of protein levels along recovery (18, 19). Furthermore, our studies provided first evidence for a regulatory role of the methionine biosynthesis pathway in response to drought, and were ground for a more detailed investigation (20). In another study (12), we found a relationship between plant nitrogen assimilation strategies, and drought responsiveness. When comparing N-fertilized with N-fixing *M. truncatula* plants we showed a nutritional priming effect that separated the initial metabolic control status of the plants such that upon drought protein and metabolite levels of the leaves showed opposite trends. Although levels of several analytes in N-fertilized plants increased, most drought responsive metabolite and protein levels of nodulated N-fixing *M. truncatula* decreased. In addition to the initial status of the plant that can be positively influenced by endophytes *e.g.* because of their growth-promoting effects, variation in molecular stress response and stress tolerance can occur depending on the degree of drought stress and stress exposure kinetics (21, 22). Plants adjust their molecular responses as drought stress increases (17). Thus, time series become more and more crucial in understanding the dynamics of molecular stress adjustment mechanisms. Transcript data, however, do not reflect the actual adjustment processes taking place at a certain time point. In fact, correlations of transcript-to-protein and thus dynamic changes in abundance are only very low (23–25). Vogel and Marcotte (25) reviewed that the major role of protein regulation occurs after transcription on the post-transcriptional, translational, and degradation level. It is therefore important to analyze the post-genomic level of molecular plasticity using comprehensive systems biology approaches that combine front-end proteomics, metabolomics and computationally assisted data evaluation techniques.

Protein turnover is an important regulatory mechanism that allows cells to respond to changing environmental conditions. To fully interpret protein abundance data from proteomic experiments, it is necessary to understand the contributions made by the opposing processes of synthesis and degradation to the transition between the states being compared. Protein translation via ribosomes as well as proteolysis through proteases or the ubiquitin-proteasome complex usually takes place in the cytosol. Thus, protein regulation is highly controlled through protein–protein interaction. This also explains, why transcript and protein changes upon environmental perturbation often do not correlate well (25). However, research on the regulation by protein synthesis and degradation is still in its infancy. Only very recently did this come more into focus (26–30). Technically, the study of protein regulation and turnover is a very challenging task. Mass spectrometry based analyses of post-translational modifications as well as metabolic labeling using ^15^N have emerged as important strategies for proteomic plasticity and turnover studies, which have been applied to a variety of organisms from yeast to *Arabidopsis* and others (26, 31). Stable isotope labeling *in planta* (SILIP)^1^ is a method that allows soil-grown plants to be efficiently labeled using a ^14^N/^15^N isotope coding strategy (32). Mass spectrometry-based protein turnover analysis based on partial metabolic labeling is still in an initial state. So far, SILAC (stable isotope labeling by amino acids in cell culture (33)) was the most common procedure for protein turnover analysis of plants (26, 34). Recently, various software tools have been developed to enable the automated extraction of ^15^N partial metabolic labeled peptide spectra (35–37).

As mentioned above, the molecular adjustment to drought stress has been investigated mainly by studies on drought and less through its recovery mechanisms. Recovery processes, however, directly reflect the molecular capacity of the plant on which stress deacclimation is based. A focus on the plant's stress-induced priming status that constitutes its ability to recover may be an important asset for the investigation of bioengineering strategies toward drought tolerant plants.

This study reveals proteome adjustments that may serve as evidence for the discrepancy between transcript and protein regulation dynamics. It is to our knowledge the first and most comprehensive study combining SILIP ^15^N partial metabolic labeling, (1) to unravel the plant's molecular stress recovery plasticity and strategy at both metabolic and proteomic level, (2) to understand in depth the dynamics of protein turnover and regulation throughout stress recovery, and (3) to identify key metabolic mechanisms to better understand plant stress plasticity and tolerance.

## EXPERIMENTAL PROCEDURES

### 

#### 

##### Plant Growth

The seeds of barrel medic (*M. truncatula* A17 cv. Jemalong) were surface sterilized and sown in pots (16 cm diameter; 800 ml capacity) containing a mixture of perlite/vermiculite, 2:5 (v:v). Plants were grown under controlled conditions in a growth chamber (14 h day and 10 h night; 300 μmol m^−2^ s^−1^ photosynthetic photon flux density; 22 °C day and 16 °C night temperatures; 50–60% relative humidity), comparable standard growth conditions as described before (12, 18, 19, 38).

During the first week of growth, plants were watered with nutrient solution (Evans, 1981) containing 0.5 mm ammonium nitrate. The following 5 weeks a nutrient solution with ammonium nitrate concentration of 2.5 mm was applied. Six week old plants were divided into two sets: (1) control plants were further watered to pot capacity with 2.5 mm ammonium nitrate Evans nutrition solution, while (2) drought stress was applied to another set of plants by water withholding for 10 days until rewatering.

##### Rewatering Experiment, SILIP, and Harvest

After 10 days of drought stress, one part of the drought stressed plants and controls were harvested. The other part of plants were again separated into subsets: (1a) control plants were further watered with **^14^**N- ammonium nitrate; (1b) control plants were changed to **^15^**N- ammonium nitrate (**^15^**N nitrate-**^15^**N, 98% **^15^**N; Sigma); the stressed plants were rewatered with either (2a) **^14^**N- or (2b) **^15^**N- labeled ammonium nitrate containing growth medium. Plants were washed two times with water before **^15^**N medium application. The growth medium was supplied daily to pot capacity 1 h after the onset of light. Rewatering was carried out for another 4 days and *M. truncatula* roots and shoots were harvested 2, 24, 48, 72, and 96 HAR, always 2 h after the onset of light. Harvests were directly frozen in liquid nitrogen and stored at −80 °C until further processing. This resulted in four sets of samples, each with five time points: (1) drought stress including the recovery phase and (2) nonstressed control; each with a ^15^N-labeled set and a nonlabeled set.

##### Physiological Parameters

The predawn xylem water potential was measured before the onset of the photoperiod during drought stress recovery periods, using a Scholander pressure bomb. From day 0 to 10 of withholding water the substrate water content during drought was estimated gravimetrically from the pot weight of control and drought stressed plants. During recovery the following parameters were measured: Stomatal conductance (g_s_) was measured 3 h after onset of the photoperiod with a steady-state porometer (PMR-4, PP Systems, Hitchin, UK) connected to the EGM-4 gas monitor, serving as data logger. Primary chlorophyll fluorescence parameters (Fm′, F′) were measured on the adaxial surface of mature leaves employing a saturation pulse method, using the MINI-head version of theIMAGING-PAM chlorophyll fluorometer M-series (Heinz Walz GmbH, Effeltrich, Germany). The PSII operating efficiency was calculated by *F*_q_′/*F*_m_′ = (*F*_m_′-*F*′)/*F*_m_′ (39). Analysis was carried out on six biological replicates for each of the previously described conditions (supplemental Fig. S1).

##### Extraction and Derivatization of Polar Primary Metabolites for GC-MS Analysis

*M. truncatula* roots and shoots were ground to fine powder using liquid nitrogen. Fifty mg of the powdered tissue were used for the extraction with freshly prepared and precooled (−20 °C) extraction buffer (MeOH:CHCl_3_:H_2_O, 2.5:1:0.5) as previously described (40). Six replicates per treatment (three biological, two technical) were randomly injected to discriminate technical from biological variation.

##### GC-TSQ-MS Settings

For metabolite profiling GC-MS is mostly the method of choice. Here we used GC hyphenated to triple quadrupole (Thermo Scientific TSQ Quantum GC™, Bremen, Germany), as previously described in detail (40).

##### Primary Metabolite Detection, Identification, and Relative Quantification

The criteria used for identification were fragmentation patterns that are characteristic for the particular compound, retention time (RT) and the retention index (RI). The identification of each analyte was achieved by matching the MS-spectra and RT against (1) an in-house library (modified gmd database http://gmd.mpimp-golm.mpg.de/download/); (2) AMDIS (calculation of retention indices and comparison with RI of compounds in the mass spectral library), and (3) matching against the in-house measured standards. Calculation of retention indices was performed using the RT of the detected compound and the RT of the RT-index marker (alkane mixture), calculated with AMDIS for representative samples of different treatments. Because of derivatization, in some cases more than one peak was detected for one metabolite. These peaks were initially analyzed separately and summed up for further analysis or data mining. About 15% of the detected analytes were identified as unknown compounds. Calculation of the peak areas was performed as described previously (40). The list of detected components and calculated areas was exported to an Excel file. We used an in-house Matlab tool to produce a complete data matrix automatically. Dry weight of each sample was determined after drying 100 mg of fresh weight from the same fine powdered sample fraction. The data matrix was normalized to the respective sample dry weights and the IS for relative quantification.

To detect and relatively quantify the ^15^N labeled amino acids, adjusted quant masses (QM) were used for the changed fragmentation patterns. The molecular weights of ^15^N amino acids increased +1 for every N contained. For all identified amino acids QM of ^14^N +1 was used. For amino acids that contain two or more N, the formula: ^15N^QM_AMINO ACIDx_ = ^14N^QM_AMINO ACIDx_+ n_N(AMINO ACIDx)_ (AMINO ACIDx = particular amino acid; *n* = number of N contained in AMINO ACIDx) was also tested and showed no significant difference to QM_14N_ + 1.

##### Extraction of Secondary Metabolites

For the structural elucidation of flavonoids 50 mg fresh weight of frozen plant material were homogenized in 1 ml of 80% methanol. The suspension was placed in an ultrasonic bath for 5 min. The extract was centrifuged (21,000 × *g*, 10 min) and the supernatant was transferred to a new eppendorf tube. Internal standard, human peptide [GLU1]FIBRINOPEPIDE B (100 μl of 1 pmol/μl) was added. Solvent was evaporated in a vacuum concentrator. The dried samples were resolved in 50 μl of 50% MeOH in 0.1% FA. The samples were centrifuged (21,000 × *g*, 10 min). The supernatant was diluted (1:10) with 0.1 FA to 5% organic constituent. Afterward, the samples were centrifuged (21,000 × *g*, 10 min) for 10 min, and at least 15 μl of samples were immediately used for LC-MS measurement.

##### nanoESI LC-MS/MS for Secondary Metabolite Analyses

Metabolites were separated with a reversed phase column (HSS T3, 1.8 μm, 100 μm × 100 mm, nanoAcquity, Waters, Milford, USA) coupled to a one-dimensional nano-flow LC system (UltiMate 3000, Thermo Fisher Scientific, Austria), using a 50 min gradient ranging from 95% solvent A (0.1% FA in water) to 90% solvent B (90% acetonitrile, 0.1% FA in water) and a flow rate of 0.5 μl per minute. For each treatment three biological and two technical replicates were randomly analyzed. MS analyses were performed in positive mode on a LTQ-Orbitrap XL (Thermo Fisher Scientific, Waltham, MA). Scan range was 100 to 900, resolution was set to 60,000 and tube lens offset 160 V. A data dependent top five MS/MS fragmentation was applied.

Compounds were identified by combining the results of two different methods. Firstly by matching the exact masses of the ionized and fragmented molecules with an in-house measured standard library and compounds from the literature. Secondly, by matching the gained MS/MS spectra with in-house measured standards and the spectra collected in the freely accessible repository named Mass Bank (41). For quantification, raw-Data files were converted to mzXML format using the MassMatrix mass spectrometric data file conversion tool version 3.9 from the Case Western Reserve University (Cleveland, Ohio; http://www.massmatrix.net/). ProtMAX version 2012 was used for data deconvolution, which allows mass accuracy precursor alignment of selected *m*/*z* signals (supplemental Table S1) and generation of a quantitative data matrix (42).

##### Protein Extraction for Shotgun LC-MS/MS Analysis

Three biological replicates were used for protein extraction. Shoot and root (200 mg of liquid nitrogen frozen fresh weight material) were extracted separately. Frozen shoot tissue was homogenized in 1 ml of urea buffer containing 50 mm HEPES, pH 7.8 and 8 m Urea using a glass homogenizer. After centrifugation (10,000 × *g*, 10 min, 4 °C) the urea soluble proteins in the supernatant were precipitated overnight in five volumes of −20 °C cold acetone containing 0.5% β-mercaptoethanol. The precipitate was pelleted at 4000 × *g*, 4 °C for 15 min. The resulting pellet was washed with −20 °C cold methanol containing 0.5% β-mercaptoethanol and again centrifuged (4000 × *g*, 10 min, 4 °C). Root tissue was TCA-phenol extracted following the protocol of (43). Air-dried protein pellets were dissolved in 500–800 μl of urea buffer (described above) and protein concentration was determined by Bradford assay using BSA as a standard.

##### In-solution Protein Digestion

In-solution digestion was performed using 100 μg of protein. Initially the endoproteinase LysC (Roche, Mannheim, Germany) was used for a first digestion (1: 100 v/v, 5 h, 30 °C). For the second digestion step, samples were diluted with trypsin buffer (10% ACN, 50 mm Ammonium bicarbonate, 2 mm CaCl_2_) to a final concentration of 2 m Urea and incubated overnight at 37 °C with Poroszyme immobilized trypsin beads (1:30, v/w; Applied Biosystems, Darmstadt, Germany). The digest was desalted with C18-SPEC 96-well plates (Varian, Darmstadt, Germany) according to the manufacturer's instructions. The eluted peptides were vacuum-dried.

##### nanoESI LC-MS/MS for Protein Analyses

Peptide digests (1 μg each) were randomly separated using a Peptide ES-18 column (15 cm x 0.1 mm, 2.7 μm; Sigma-Aldrich) coupled to a one-dimensional nano-flow LC system (UltiMate 3000, Thermo Fisher Scientific, Vienna, Austria), using a 90 min gradient ranging from 95% solvent A (0.1% FA in water) to 80% solvent B (80% acetonitrile, 0.1% FA in water) and a flow rate of 0.4 μl per minute. For each treatment three biological and two technical replicates were randomly analyzed. MS analyses were performed on a LTQ-Orbitrap XL (Thermo Fisher Scientific). For the database dependent spectral count analysis (Wienkoop, 2011), a top 7 MS analysis setting was used with the full scan range from 350 to 1800 *m*/*z*. Dynamic exclusion settings were as described in Hoehenwarter and Wienkoop (44). Briefly, repeat count was set to 1, repeat duration 20 s, exclusion list size 500, exclusion duration 60 s and exclusion mass width 10 ppm. Charge state screening was enabled with rejection of unassigned and 1+ charge states. Minimum signal threshold counts were set to 1000. Notably, for better performance of the ^15^N-labeling analysis the resolution was set to 60,000. Replicates per treatment (3 biological, 2 technical) were randomly injected to discriminate technical from biological variation.

##### Protein Identification and Relative Quantification

We used the SEQUEST algorithm and Proteome Discoverer (v 1.3, Thermo Scientific) to search MS data against a composite protein-fasta-file, which was created by fusing the following three databases:

1. Uniprot UniRef100 Medicago, origin: www.uniprot.org, Uniprot advanced-search *M. truncatula* [3880], UniRef100. The search was performed on May 7th 2013 and resulted in 54,246 entries.
2. IMGA, origin: http://medicago.org/genome/IMGAG/. 64,123 entries.
3. DCFI origin (no longer available): http://compbio.dfci.harvard.edu/tgi/cgi-bin/tgi/gimain.pl?gudb=medicago. 412,908 → 68,818 entries.

From the nucleotide database, a protein-fasta-file was translated using Emboss (resulting in 412,908 entries). Thereof only the longest ORF per accession number was selected using an unpublished in-house Python script (resulting in 68818 entries). The three fasta files described above were combined, producing a new nonredundant fasta-file containing 130,824 entries.

In-silico peptide lists were generated with the following settings: trypsin as the digestion enzyme, a maximum of three missed cleavages and methionine oxidation as dynamic modification. Mass tolerance was set to 5ppm for precursor ions and 0.8 Da. Additionally, a decoy database containing reversed sequences was used to estimate the false discovery rate (FDR). Known contaminants have been excluded. Only high confidence (FDR ≤ 1%) peptide identifications with a minimum XCorr of 2 and proteins with at least two distinct peptides were considered. Protein relative quantification is based on unique peptide specific spectral counting (SC) as described previously (12). Proteins were used for quantification when SCs were in 6 of 6 replicates of at least one treatment and with at least 2 counts of at least 2 proteotypic peptides.

A Mapman mapping file was created on the basis of the in house fasta file using Mercator (45, 46).

##### Statistical Significance and Functional MapMan Cluster Analyses of Physiological, Metabolite, and Unlabeled Protein Data

**Fig. 1.**
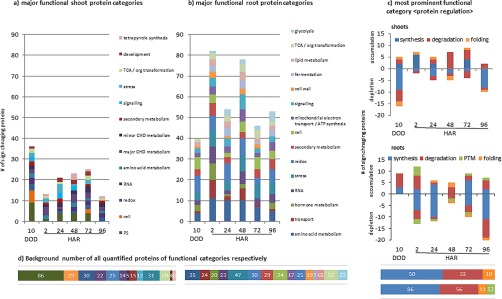
**Major functional protein categories (MapMan) of *M. truncatula* shoots and roots, given as number of proteins significantly altered in abundance (one-way ANOVA *p* < 0.05; *n* = 3) after 10 days of drought (10 DOD) and during the course of rewatering.** HAR = hours of rewatering. The major category protein regulation is shown separately and distinguishes numbers of accumulating (positive numbers) and depleting (negative numbers) proteins compared with control of protein-synthesis, -degradation, and -folding. For roots additionally – the subcategory posttranslational modification (PTM). Functional groups are only displayed if number of proteins > 4 in at least one time point. Information taken from supplemental Table S2. Background from all quantified proteins (ID in all six replicates of at least one treatment).

**Fig. 2.**
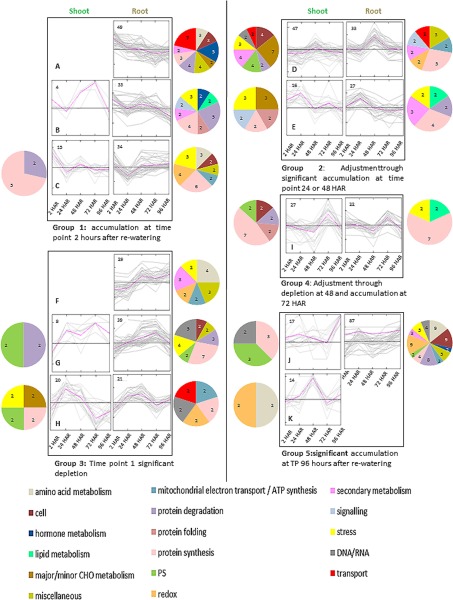
**Correlative cluster analysis (K-means, A–K) of protein abundance from log transformed ratio (treatment *versus* control) showing significant changes in response to drought-recovery across five time points (ANOVA *p* ≤ 0. 05).** Clusters were further grouped according to pattern similarity of response. Numbers of proteins are showen in each cluster. Pie charts of main functional groups of proteins (≥ 2 proteins) are represented corresponding to clusters (left for shoot, right for root).

By calculating ratios between control and treated samples the physiology, as well as metabolite and protein data, were analyzed in detail. Significant differences between these were determined using Student's *t* test at *p* < 0.05 for physiological (Supplemental Fig. S1) and labeled metabolite data (Fig. 4). The statistical significance of protein and metabolite abundance changes among treatments were evaluated by one-way ANOVA using TukeyHSD (R-Studio v3.2.2 (47)). Only proteins and metabolites with *p* < 0.05 were considered for further analyses. Note: We decided for a one-way ANOVA extracting the significance of treatment *versus* control for each specific TP to reduce effects that occur during diurnal regulation and development. Hence, we do not show nested significance of the ratios over time. Hierarchical clustering heatmaps for roots and shoots under drought (D) and drought-recovery (DR) with details on fold changes, *p* values, functional annotations (GO and MapMan), and others are given in supplemental Table S2. Significantly, changing metabolites and proteins were further used for correlative clustering analysis (Mev software v4.9) by k-means with 11 clusters (A-K) (Fig. 2). MapMan functional categorization was used for overrepresentation in Figs. 1 and 2.

##### Principal Component, Correlation Network, and Functional GO Cluster Analysis

**Fig. 3.**
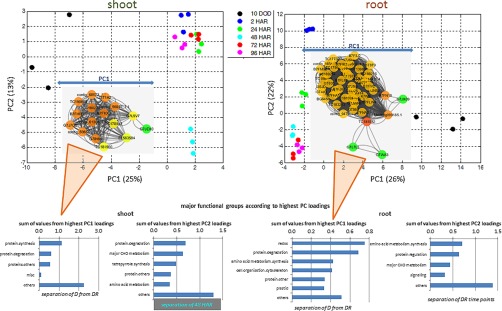
**PCA of significant changed root and shoot protein and metabolite (treatment to control) ratio data.** Inserted Functional Correlation Network Analysis (cytoscape) based on highest loadings (< −0.9 and > 0.9 as of PC loadings supplemental Table S3); Nod color = Edge-Betweenness increases from green to red. Sum of loading values of major functional groups (> three proteins).

For significantly changed proteins and metabolites during drought and recovery, Principal Component Analysis and subsequent Correlation Network Analysis (Fig. 3) calculations were carried out using the Matlab tool Covain 12-03-22 (48) with the following settings: Correlation time series; Spearman correlation coefficient 0.5. For visualization, the resulting .sif files were uploaded to Cytoscape 3.2.1 (49). GO annotations for the proteins were retrieved and visualized from AgBase 2.00 (50) tools GoRetriever and GOSlimViewer (supplemental Fig. S2i-2iii). This was however, restricted to accession numbers from UniProt and IMGA. Lists of GO annotations can also be found in Supplemental Table S2; PCA loading information (Supplemental Table S3).

##### Protein Turnover Calculations of ^15^N Labeled LC-MS/MS Protein Analyses

Selected Peptide Extraction list (SelPEx; (51)) generation, turnover calculation (37) and data filtering procedures are described in detail in the Supplemental information file “Selpex and Turnover”). Protein turnover is calculated by the Relative Isotope Abundance (RIA), which is defined as the ratio between the intensity sum of all heavy (^15^N) to the intensity sum of all light (^14^N) and heavy peaks (as described in detail in (34)).

This procedure resulted in turnover data for 657 proteins and 3076 peptides for shoots and 428 proteins and 1448 peptides for the roots (supplemental Table S4).

These data were used for the following statistical visualizations using the “prcomp” and “ggplot2” library in R: Principle component analysis of relative isotope abundances (RIAs) (supplemental Fig. S3), Hierarchical cluster analysis (Euclidean distance) of RIA ratios drought-recovery *versus* controls (DR/C) (Fig. 5) and Boxplots of putative marker proteins (Fig. 6).

## RESULTS

### 

#### 

##### Physiological Response of M. truncatula to Drought and Recovery

To study the effect of reduced water availability and subsequent rewatering we first induced a gradually increasing drought stress by water withholding. As a result, the substrate water content in pots of treated plants decreased strongest during the first 6 days of the experiment (supplemental Fig. S1*A*). Subsequently the desiccation rate of the substrate (here the Vermiculite:Perlite mixture) declined and plants showed first signs of wilting. At day 10, the ultimate time point of the drought stress experiment, the SUBSTRATE WATER CONTENT dropped to 40% of the pot water holding capacity. This result indicates severe drought observed by *M. truncatula* plants 10 days after water withholding.

The predawn xylem water potential is a good proxy for the water potential in the substrate solution. At day ten, drought treated plants had a mean predawn xylem water potential of −1.7 MPa concomitant with a significant decrease in stomatal conductance (supplemental Fig. S1*C*). This is commonly classified as severe drought stress because the predawn water potential of the xylem indicated that the substrate water potential was below −1.5 MPa (*i.e.* the permanent wilting point), an energy state at which the extraction of water from the soil solution is generally considered to be inhibited. The recovery-phase from severe drought-stress was initiated with rewatering the pots to 100% pot capacity (see Rewatering Experiment, SILIP, and Harvest). After two hours the water potential of drought stressed shoots nearly reached control levels (no significant difference between treatment and control, supplemental Fig. S1*D*). The stomatal conductance was restored 96 h after rewatering (HAR). A significant decrease in PSII operating efficiency between control and stress-treated plants was measured 24 h before rewatering and at the onset of rewatering (0.6 and 0.52 Fq′/Fm′, respectively) reaching the control levels again 48 HAR (supplemental Fig. S1*B*). Altogether, physiological parameters indicate a full recovery at 96 HAR (supplemental Fig. S1).

##### Functional and Quantitative Analysis of Root and Shoot Proteins and Metabolites Involved in Drought Stress Acclimation

Altogether, 1162 unique proteins (supplemental Table S5 including information in ID quality and also available through PRIDE repository with the data set identifier PXD001728), 88 different metabolites of the primary metabolism (GC-MS analysis, according to (40)) and 14 different metabolites of the secondary metabolism (LC-MS analysis, supplemental Table S1) were identified.

We first analyzed the response status of proteins and metabolites at severe drought stress after 10 days of drought (10 DOD). About 156 (11%) of all identified proteins and 20% of all identified metabolites changed with high significance upon severe drought stress comparing treated with control plants (one-way ANOVA, *p* < 0.05; *n* = 3) in shoots and roots altogether (supplemental Table S2).

Primary metabolite levels were slightly more affected in roots (12) than in shoots (8) in terms of numbers. Altogether all metabolites were found depleted except for the stress marker proline (threefold accumulation) in roots, and pinitol (twofold accumulation) in shoots. Both in roots and shoots amino acids were the major group of changing metabolites followed by carbonic acids (TCA-cycle). Sugar alcohols (minor CHO metabolism) but no sugars (major CHO metabolism) were found to significantly change in the severe drought samples. Changes of the amino acids (AA), organic acids and CHO metabolism upon long term drought stress have previously been reported for Medicago (17). In the nodule free roots and shoots AA levels decreased, in contrast to our previous findings of the root nodules (19). However, these results are in good accordance with comparative studies previously conducted on roots and shoots of N-fixing and N-fertilized plants showing differential metabolic and proteomic regulation upon drought (12). Nevertheless, differences among studies are very common (9, 12, 17). Besides differential stress conditions, this might also be because of plants developmental stages and cultivars that vary between the studies and might have an effect on the leaf senescence process during drought (52). Taken together, proline and pinitol are among the most robust and reproducible metabolic drought stress markers.

Protein response to severe drought also seemed more pronounced in roots than in shoots in terms of the extent of fold changes. However, a similar number of about 11% for both, root and shoot proteins were found significantly changed upon severe drought stress (Table I*A*). From shoots 23 of these were accumulated whereas 57 proteins were depleted. In contrast, in roots all drought responsive proteins (76) were accumulated compared to controls and none of those occurred depleted. Among those root proteins, 14 (21%) were assigned to “protein regulation” followed by “redox” (8), “cell” (6), “amino acid,” “stress,” and “miscellaneous” with 5 to 6% and several smaller categories (Fig. 1*B* and supplemental Table S2). From the root proteins of the “protein regulation” category all were accumulated, about 66% being involved in protein degradation, where most of them were subunits of the proteasome (Fig. 1*C*). The shoot responsive proteins were also largely represented by the functional category “protein regulation” (26%) (Fig. 1*A*). Among these, 6 (26%) were involved in degradation, represented mainly by proteases and the proteasome. Another 11 (48%) were involved in synthesis, mostly by depletion of eukaryotic ribosomal subunits. The results show a regulation of the proteome that can be differentiated in synthesis and degradation. Especially in roots, accumulation and enhanced presence of ubiquitin proteasome system (UPS) proteins with increased cysteine protease levels and proteins involved in synthesis (ribosomal proteins) imply differentially regulated synthesis and degradation processes.

**Table I:** Proteins of M. truncatula shoots and roots A) significantly altered in abundance (one-way ANOVA *p* < 0.05; *n* = 3) after 10 days of drought (10 DOD) and during the course of re-watering. HAR = hours of rewatering. B) Proteins, overlapping in significantly altered abundance (one-way ANOVA *p* < 0.05; *n* = 3), comparing two sequential time points respectively 10 DOD to 96 HAR. swap = overlapping change with opposite direction; up = accumulated protein abundance; down = decreased protein abundance.

A						
Shoot	10 DOD	2 HAR	24 HAR	48 HAR	72 HAR	96 HAR
Up	24	13	24	40	24	9
Down	56	16	19	15	24	25
Sum all	80	29	43	55	48	34
Root	10 DOD	2 HAR	24 HAR	48 HAR	72 HAR	96 HAR
Up	76	80	46	86	52	51
Down	0	64	57	45	35	58
Sum all	76	144	103	131	87	109
B						
Shoot	10 DOD-2HAR	2–24 HAR	24–48 HAR	48–72 HAR	72–96 HAR	
Up	0	2	5	4	0	
Down	2	2	0	0	5	
Swap	1	4	6	9	10	
Sum all	3	8	11	13	15	
Root	10 DOD-2HAR	2–24 HAR	24–48 HAR	48–72 HAR	72–96 HAR	
Up	7	13	13	21	11	
Down	0	12	14	10	11	
Swap	4	18	15	13	11	
Sum all	11	43	42	44	33	

Other major drought-responsive shoot proteins were from “photosynthesis” and “cell” followed by several categories with less than 6 proteins per unit (Fig. 1).

In addition, protein data show the sensitivity of both shoots and roots to drought-induced stress like oxidative damage manifested through significant induction of ROS proteins, such as peroxidase, superoxide dismutase, ascorbate reductase, alcohol dehydrogenase, etc. (supplemental Table S2). Such induction of ROS is well documented and in accordance with existing literature (53). In roots we identified a higher number of redox proteins involved in drought stress response than in shoots (Fig. 1), most of them showing a significant increase in abundance compared with the control levels. In general, the response on the root level shows main differences to shoot levels in terms of functional categories.

The categories photosynthesis (PS), major and minor CHO metabolism, development and tetrapyrrol synthesis are mainly present in shoots whereas transport, hormone metabolism, cell wall, mitochondrial electron transport and ATP synthesis, fermentation, glycolysis and lipid metabolism are exclusively dominating the response of the root proteome (Fig. 1). Noteworthy, only in roots hormone metabolism proteins mainly represented by lipoxygenases also involved in jasmonate biosynthesis were significantly accumulated during severe drought (supplemental Table S2). Notably, several proteins of the biological-process group “stress” or “defense response” are uncharacterized proteins whose functional annotation has thus far only been inferred through the automated Mercator or GO annotation, respectively (*e.g.* I3SYR8, G7L8F8, and others). Hence, those data may help improve the Medicago genome annotation.

Among the secondary metabolites, in roots, no statistically significant changes were found. In shoots, however, genistein was found accumulated at severe drought stress, (supplemental Table S2).

##### Functional Cluster Analysis of Drought Stress-Recovery Responsive Metabolites and Proteins of M. truncatula Shoots and Roots

About (442) 35% of all identified proteins and (29) 56% of all identified metabolites changed significantly during 96 h of the stress-recovery progress when comparing treated with control plants in shoots and roots altogether (*p* ≤ 0.05, in at least one time-point; *n* = 3).

Less than 50% of the primary metabolites that changed during drought stress are also responsive during the recovery process (supplemental Table S2). The levels of most metabolites including proline (root) and pinitol (shoot), the two major stress responsive metabolites, went back to control levels already 2 HAR along with all other drought responsive amino acids. This indicates a rapid recovery-response of these metabolites occurring within 2 HAR. Furthermore, instead of amino acids, the major group of responsive metabolites were now sugars and organic acids of the TCA cycle as well as sugar alcohols. In the 96 HAR phase, all metabolites returned to control levels (supplemental Table S2).

In numbers of significantly changed proteins, roots showed a much stronger deacclimation response with 233 proteins compared with 140 in shoots (supplemental Table S2). Also the average fold-change seemed more pronounced in roots than shoots (more than 1.3 times higher) (supplemental Table S2). The overlap of significantly responding proteins between severe drought and the first two hours after rewatering is very low (Table I*B*). Only 3 shoot and 11 root proteins are still significantly changed at the early time points of recovery. In fact, a rapid change of the 76 drought accumulated proteins in roots was observed of which only 11 remain 2 HAR, whereas at the same time a complete new set of more than 70 proteins accumulated (Table 1*B*). In contrast, a higher number of overlapping proteins over subsequent time points of recovery especially in roots was found (Table 1*B*). Remarkably, although the category “protein regulation” was found to be the largest in drought as well as during recovery response in both, roots and shoots, only 2 of these proteins (2 out of 140) were common between stress and recovery. Overall, the data indicate independent response mechanisms uncoupling drought from recovery.

Among the significantly changing recovery proteins most belong to the functional categories “protein regulation,” “amino acid metabolism,” compared with stress also “transport” and “glycolysis” and in shoots additionally “tetrapyrrol synthesis” (Fig. 1). Relative distributions of this category across all time points are displayed in Fig. 1*A*, *B*). The largest responsive functional category “protein regulation” (∼50%) was analyzed separately (Fig. 1*C*). Here, the subcategories “synthesis,” “degradation,” “folding,” and “post-translational modification” (> four proteins in at least one-time point) were built according to the mapping file and are further presented according to their direction of relative changes.

Proteins within the functional categories “redox” and “stress” in leaves seemed to recover fastest from drought. They significantly accumulated during severe drought stress but no longer 2 HAR (Fig. 1*A*). However, the majority of proteins shared between stress and recovery response, occurred within the first time point of 2 HAR. For instance, the root ATP transporter G7KW90 as well as the GTP binding protein B7FH02 involved in transport and signaling, were found to be relevant. This supports the assumption that protein transport mechanisms are among those responding to severe drought and are also highly relevant for regulatory processes during initial stress recovery. Remarkably, in shoots most of the significantly changing proteins at TP 48 HAR accumulated. This might be related to the formation of new leaves occurring at that TP.

To test time dependent correlation of those functional groups, proteins were hierarchically clustered along the temporal gradient of stress recovery over 5 time points (TP) and depending on the direction of change(s) using the k-means of Covain (48) (Fig. 2, supplemental Table S2). Clusters (A to K) were further assembled into 5 regulatory groups of close correlation as follows: *group 1*) accumulation at TP 2 h after rewatering; *group 2*) accumulation at TP 24 or 48 h after rewatering; *group 3*) significant depletion at TP 2 h after rewatering; *group 4*) significant depletion at TP 24 or 48 h after rewatering, and *group 5*) accumulation at TP 96 h after rewatering. Thus, a negative correlation between group 1 and 3 as well as 2 and 4 might exist. Fig. 2 describes the distribution of the main functional protein groups of the correlation clusters for shoots and roots, respectively.

Even though most of the MapMan functional groups are also distributed across correlative clusters, no clear correlation of single functional groups seems to exist, as anticipated from the analysis of distribution over time points. This supports the hypothesis that several pathways are interconnected during the process of recovery.

However, protein synthesis seems more pronounced in the correlative group 1 (accumulating 2 HAR) compared with group 3 (depleting 2 HAR). Interestingly, the major functional categories of correlation cluster A, are transport and hormone metabolism.

##### Correlative Network Analysis (CNA) of Proteins and Metabolites and Functional GO Network Analysis Based on Principal Component Analysis (PCA)

Although ANOVA statistics allow for the determination of significantly changed proteins/metabolite levels related to time point (TP) specific controls, principal component analysis (PCA) allows for visualization of TP separation and the detection of analytes involved in their separation.

For roots and shoots, the main networks were selected, corresponding to proteins and metabolites with the highest impact on separation of drought *versus* recovery (supplemental Fig. S2). Not surprisingly, the PCA revealed a clear separation between drought and recovery as well as a correlative network such that the highest PC loadings (supplemental Table S3), separating drought from recovery processes, were directly linked to most of the significant drought responsive proteins and metabolites. Major functional groups according to MapMan are in a) shoots: protein regulation (mainly synthesis and degradation); in b) roots: redox, protein degradation, amino acid metabolism and cell organization.

This was further confirmed by a functional GO network analysis of those proteins, which revealed major biological processes (1) in shoots: cellular processes and component organization as well as metabolic processes leading to protein metabolic processes and (2) in roots: response to stimulus followed by cellular component organization and developmental processes (Fig. 3 and supplemental Fig. S2ii).

In comparison, the functional MapMan analyses of the correlative recovery network revealed (1) in shoots: (PC 2), a pronounced separation of TP 48 h after rewatering involving mainly protein regulation, major carbon metabolism, tetrapyrrol synthesis, and amino acid metabolism and (2) in roots: (PC1) a clear separation of TP 2 h after rewatering from the other TPs also showing amino acid synthesis, protein regulation, major carbon metabolism and signaling putatively with strongest impact on division (Fig. 3).

##### Relative Amino Acid Turnover Analysis

**Fig. 4.**
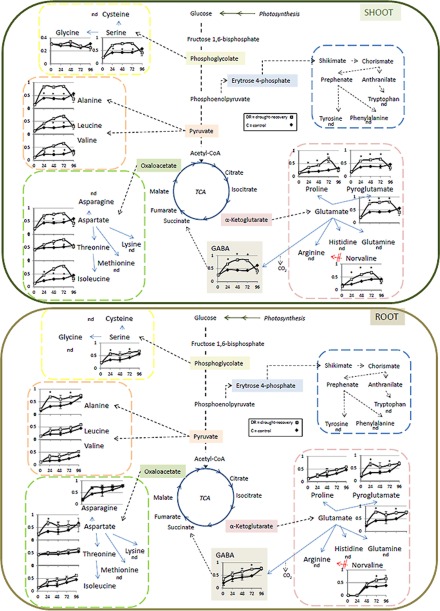
**Time dependent change of amino acid turnover rates during drought-stress recovery in *M. truncatula* shoots and roots**. The *y* axis represents the turnover rate expressed as relative isotope abundance (RIA), the *x* axis shows the time after rewatering in h. Values are means ± S.E. (*n* = 3). * indicate student's *t* test significance (*p* < 0.05). Open square = drought-recovery (DR); closed diamond = control (C).

Amino acids are the metabolites that incorporate N via uptake of nitrate or ammonium during N-assimilation (54). The relative isotope abundance (RIA) describes the abundance of the natural isotopic envelope/abundance ^14^N in relation to the entire isotopic envelope/abundance consisting of the natural, as well as the ^15^N enriched part of the spectrum ^15^N (^15^NNA). RIA_0_ (^14^N/^14^N+^15^N_NA-_ also L/H+L) is usually constant and was found in this study in controls and treated *M. truncatula* shoots and roots between 0.8 and 0.9. Only, RIA_0_ of isoleucine was lower with 0.6, meaning a higher relative incorporation of ^15^N_NA_ for this amino acid (Fig. 4). When calculating RIA for the ^15^N metabolic labeling experiment, the ratio H/H+L was used to determine the ^15^N incorporation rates (Fig. 4). In control shoots the average RIA of about 0.1–0.2 (in accordance with RIA_0_ of 0.8–0.9) 2 h after ^15^N supply increased up to 0.3–0.6 and in roots up to 0.8 at 96 h of ^15^N supply. Amino acid RIAs of drought-stressed plants reached these levels already within 24 HAR. In shoots the RIAs of most amino acids reached even higher maxima (up to 0.9) at this early time point (alanine > glutamate > aspartate > gaba > serine) or 72 HAR (threonine > isoleucine > leucine > valine > proline). This result implies a rapid amino acid synthesis in the first 24 HAR in accordance with increasing amino acid levels along the time course compared with controls. Most rapid leaf amino acid turnover rates have previously been reported for alanine (55). In roots ^15^N incorporation rates into amino acids from 2–24 HAR was asparagine > alanine > glutamine > aspartate > serine. Asparagine, glutamine and aspartate together with glutamate are the four major amino acids in plants that translocate organic nitrogen from source to sink (56). Hence, the data suggest an increased allocation of these amino acids from roots. However, there are no indications that these metabolites are more enriched in our protein data set.

##### Relative Protein Turnover Analysis

**Fig. 5.**
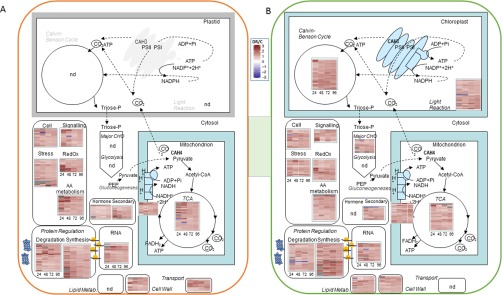
**Functional overview of protein turnover data as a function of relative isotope abundance (RIA) ratios (DR/C) of *A*, roots and *B*, shoots.** RIA ratios derived from our automated software tool (37) are presented as heat maps visualized using “ggplot2” in R. Red indicates higher and blue indicates lower drought-recovery (DR) to control (C) ratios, respectively. A full list of data and statistics are given in supplemental Table S4.

Synthesis and degradation of proteins involve the incorporation and release of proteinogenic amino acids. Thus, a protein turnover analysis using partial ^15^N metabolic labeling comprises the ^14^N/^15^N amino acid ratio analysis as well as the mass spectrometric analysis of the incorporation of labeled amino acids into peptides. These values do not directly reflect turnover rates because they underestimate degradation of heavy ^15^N species and hence the actual synthesis/degradation rate (36). They do also not reflect the % of ^15^N incorporation (57, 58), a complex calculation process that is not available in an automated manner and thus not implemented here. Nevertheless, RIA also called q-value has often been used as the basis for turnover calculations (59, 60). Here, we used RIAs, data on ^14^N and ^15^N abundances and compared changes over time as well as protein fold changes for the estimation of relative turnover dynamics. Similar to Martin and colleagues (36), we calculated synthesis and degradation rates using light (^14^N) and heavy (^15^N) peak intensities (Supplemental Table S4 and Supplemental information on calculation of Kdeg and Ksyn). PCA of RIA values revealed a clear separation of C and DR over time (Fig. 5). PC1 shows a continuous change in ^15^N incorporation rates that is more pronounced than increasing differences over time between D and DR. This is because of the fact that especially at time point 2 HAR, no incorporation and thus no differences between the treatments were observed.

**Fig. 6.**
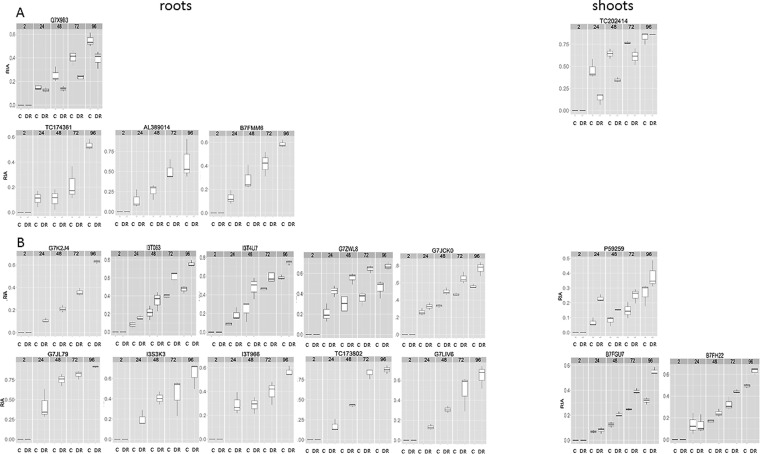
**Boxplots of putative marker proteins involved in the regulation of drought-recovery (DR).** These proteins showed significant changes (DR/C) in protein abundance and RIA levels. Boxplots show RIAs of (controls) C and (drought recovery) DR along the different time points (TPs), 2 to 96 h after rewatering. *n* = 3

Furthermore, even though generally a stronger increase in RIA of DR treatment compared with control is visible, not all proteins follow this trend (Fig. 6). HIC analysis on RIA ratios for DR *versus* control (C) combined with functional cluster analysis was carried out. An overview for roots and shoots is given in Fig. 5. Overall, the drought recovery *versus* control RIA ratios show a higher ^15^N incorporation during drought recovery (red color). This observation is explained by an overall stronger isotope incorporation into proteins of drought treated samples. It does not necessarily indicate higher synthesis rates but in most cases it shows a higher incorporation through increased availability of ^15^N-labeled amino acids directly after rewatering compared with the control plants. This also indicates a faster ^15^N-containing water/nutrition uptake after the period of water deprivation. Especially in roots, an increased RIA drought recovery (DR) to control (C) ratio can be observed in the first 24 to 48 HAR, indicated by a higher ratio (Fig. 5). Specifically, the functional categories RedOx, amino acid metabolism and protein regulation show the highest values. In the shoots this early response is less pronounced, however, the functional group “protein synthesis” reveals high RIA ratios (DR/C) along the time course of recovery. In roots but also shoots most RIA ratios flatten and thus come closer to control levels. This trend toward abating of increased ^15^N incorporation and re-organization at 96 HAR is underlined by principal component analyses (PCA) (supplemental Fig. S3i). On the other hand, RIA ratios of most photosynthetic proteins revealed a trend to increase toward the end of the recovery phase measured (Fig. 5).

Loadings of PC2 allowed the discrimination between treatments (supplemental Fig. S3ii). In both roots and shoots the highest loadings were within the categories protein regulation, followed by stress. In shoots photosynthesis (PS) needs to be mentioned as well being among the highest loadings.

Some proteins were selected for Boxplot analysis, when statistical significance was found for both, RIA ratios and relative abundance changes (DR/C) (Fig. 6). Significant change in RIA were not always (92 out of 405 shoot- and 85 out of 259 root proteins, supplemental Table S4) connected to a significant change in protein abundance. This might reflect complexity and concerted regulation between protein synthesis and degradation. As explained above, higher ^15^N incorporation infers higher synthesis rates during recovery. Taken together with the much lower number of increasing protein levels, it is an indication for a simultaneous increase in degradation and thus in the overall protein turnover. Nevertheless, certain proteins only showed ^15^N incorporation either in controls or treated samples and for some proteins RIAs only increased at certain time points (Fig. 6), underlining the complexity of protein turnover regulation.

## DISCUSSION

### 

#### 

##### Distinct Molecular Regulation Mechanisms between Drought Stress Acclimation and Recovery Adjustment

Plants have evolved manifold strategies to respond to fluctuations in water availability. Thus, the response strategy of a plant when facing drought is related to a number of biotic and abiotic parameters (12, 21, 61). Among these parameters, stress exposure kinetics belong to the main factors constraining the comparability of studies addressing plant responses to water deprivation (62). It is well known that PS and cell growth are among the primary processes to be affected by drought (4, 63). Continuous water deficit induces early leaf senescence in plants and during this process, chloroplasts are degraded and photosynthesis drastically drops. In general, no typical leaf senescence mechanisms were detected on the protein level such as accumulation of members of the glutathione S-transferase and quinone reductase as described by Hebeler and colleagues (64). This might be because of the fact, that total shoots and mostly vital and not dried-up leaves have been extracted. As a result, dilution or even elimination of leaf specific and especially leaf senescence-specific signals might occur—if these are not highly abundant. It is likely that senescence is a sequential process, in the old leaves and the assimilating processes shut down earlier than in the younger leaves (52). As described in detail in the results, however, several other typical stress responsive processes such as the induction of ROS and phytohormone metabolism have been observed. Among those, accumulation of lipoxigenases in roots might hypothetically be an indirect indicator for jasmonate accumulation and an induction of the signaling pathway in roots. Though, jasmonate levels have not been measured and its accumulation remains speculative. Nevertheless, jasmonate is one of the phytohormones known to be involved in signaling of several biotic but also abiotic stress induced responses in leaves. More in accordance to our data however, Grebner and colleagues (65) described jasmonate accumulation in roots upon drought stress. Besides other phytohormones found to be induced upon drought stress, such as ABA and auxin, jasmonate seems the most dominant in roots, underlining the above mentioned hypothesis.

Suboptimal water supply leads to a rapid inhibition of shoot growth (66) and as expected, proteins and metabolites involved in PS, cell development- and amino acid metabolism are also among the largest drought stress responsive groups in leaves (Fig. 1). Strong cell-wall regulatory responses such as tubulin accumulation upon stress have been described before (12, 67).

Despide the knowledge about all these rather general and reproducible stress response mechanisms, including the robust markers proline and pinitol, there is only little breakthrough in improving drought tolerance.

Interestingly, not much is known about the molecular regulation during drought-recovery in legumes (10, 17, 19) or specifically about long-term summer drought-recovery, as it is common in the Mediterranean region (68, 69). In nature periods of drought are usually alternating with rain. Plants therefore need to be equipped with a highly adaptive molecular capacity of drought tolerance to enable continuous growth.

Noticeably, the present study reveals that recovery differs considerably from drought acclimation and is not only a reversible process to control conditions (Table I, Fig. 1, and supplemental Fig. PCA S2i): After rewatering, physiological, metabolic and proteomic data showed a clear recovery along a period of 96 h. Interestingly, in the course of stress recovery, the set of responsive proteins and metabolites differs significantly from that of drought stress (Table I). Although, most drought responsive proteins went back to control levels directly after rewatering, a broad new set of root and shoot proteins changed. These results are indication of independent regulatory mechanisms for drought and recovery. This might suggest that important mechanisms have been overlooked in the past and thus hampered success of smart breeding strategies. We present novel, as yet not evidenced, subsequent phases of adjustment and proteins/metabolites and their associated functional groups involved (Fig. 1). We detected strong changes in numbers of proteins in terms of abundance as well as turnover. In fact, the combination of different techniques in this study reveals complex regulatory dynamics that have not been shown before and are discussed in the following.

##### Protein Networks Functionally Involved in Protein Synthesis and Degradation Are Key Players Necessary for the Molecular Adjustment of Drought-Stress-Recovery

Along the time course of recovery, proteins and metabolites were functionally annotated and response patterns were clustered (Figs. 1 and 2). The data show a restart of the protein synthesis apparatus as well as a choke of degradation in shoots (Fig. 1*C*), regulated on ribosomal level directly upon rewatering (2 HAR) (supplemental Table S2). This emphasizes *de novo* protein synthesis to initiate biochemical processes in leaves, needed to orchestrate an appropriate stress recovery response different from stress response (70). This is for instance underpinned by an induction of proteins involved in tetrapyrrol synthesis in shoots (Fig. 2*A*).

It comprises remobilization as well as removal mechanisms of proteins that have been damaged during drought stress *e.g.* by oxidation (71). This theory is best underlined in roots by the almost complete exchange of accumulating proteins from drought to recovery and by the extraction of major functional groups with highest impact on the separation of drought and recovery in roots: redox, protein degradation, amino acid metabolism and cell organization (Table I, Fig. 2*B*, 2*C*, and Fig. 3).

This further suggests an important regulatory mechanism that is based on protein- rather than on transcriptional regulation mechanisms (25). Additionally, it shows that protein regulation is a fast process that occurs within hours and is not only explained by post-translational modifications (PTMs) but also by the induction of the protein synthesis apparatus. Interestingly, this study reveals that regulation through PTM can to some extend be detected and quantified without the need to exactly identify specific PTMs like phosphorylation, ubiquitination and others by additional enrichment strategies.

The importance of this mechanism for the plants' survival potential is underlined by the turnover data. A significant ^15^N uptake (increased relative isotope abundance) was only observed 24 HAR but not at the early time point 2 HAR. These data give evidence to the fact that new synthesis of regulatory proteins at the early stage, 2 HAR, is only possible through an amino acid allocation capacity of the plant remaining during stress and before new N-uptake. A lag-phase from N-supply until incorporation into protein is expected as it involves the whole pathway of N-assimilation from uptake to amino acid synthesis and has been found in a recent study on barley leaves to take about 9 h (29). Nevertheless, relative isotope abundance data also imply an increased *de novo* synthesis of the translational apparatus especially at early stages (24 HAR) compared with control (supplemental Fig. S3i). Thus, the metabolic capacity of the plant in terms of internal nitrogen and thus amino acid availability, prior to stress, might be a good marker for its stress tolerance, because it is necessary to re-establish an optimal metabolic state. This hypothesis is well supported by the finding that the initial N nutritional level of plants exhibit a strong impact on the sensitivity to water removal (72).

In contrast to the full recovery of metabolite levels, a set of proteins appeared to further necessitate adjustment to maintain metabolic levels. This became obvious when another set of proteins accumulated at the last stages of the recovery period. Most of these proteins functionally belong to the protein synthesis and degradation mechanism. This finding implies that some of the regulatory proteins important for initial stress-recovery adjustment may be degraded in a final step, when physiologically the plant has already fully recovered, to reach and maintain optimal metabolism.

##### Enhanced Amino Acid and Protein Turnover upon Initial 24 h Phase of Drought-Recovery and Putative Markers of Regulatory Relevance for Drought-Recovery and Stress Tolerance

Compared with previous studies where ^15^N metabolic labeling was mainly applied to cell cultures (26), increase in RIAs *in Planta* appear significantly slower, suggesting a putative overestimation of previous turnover calculations for artificial systems like cell cultures. To draw physiologically relevant conclusions especially upon environmental perturbations the importance of *in Planta* experiments for the accurate evaluation of turnover processes becomes evident. However, there may be several draw-backs such as dilution effects of ^14^N and ^15^N in the root N-uptake environment of the plant as well as inside the plant cells. Nevertheless, increased RIA ratios of drought-recovery to control, unambiguously show a differential uptake and incorporation of ^15^N into drought stressed plants when rewatered especially in the first 24 HAR (Figs. 3–5).

As described in detail in the results, N-uptake and amino acid synthesis seemed increased for drought treated plants in the initial phase of recovery (Fig. 4). These data support that within the first 24 HAR most proteins have higher turnover rates than amino acids and proteins of control plants. All amino acid RIA levels of the drought-recovery (DR) treatment go back to control (C) levels latest 96 HAR (Fig. 4). Furthermore, most RIA DR to C ratios do not change significantly between 72 and 96 HAR. These data indicate a phase of adaptation/recovery that is also visualized in the principal component analysis (supplemental Fig. S3i). Thus, even though protein RIAs are higher in DR compared with C plants the general protein turnover might no longer be increased in the late recovery state. An implicit steady-state of the present dynamic system is however not applicable.

Despite the overall trend of increased drought-recovery RIAs compared with controls, a few proteins show reduced RIA ratios upon stress recovery (Fig. 5). This effect may be expected when protein *de novo* synthesis of specific pathways has been slowed down during drought-recovery. This can be clearly shown for instance on the basis of the stress responsive protein (Q7X9B3) involved in the jasmonate biosynthetic pathway in the roots as described before (Fig. 6*A*). In leaves, a putative indicator for a shutdown of protein degradation mechanism during initial phase of DR can be found (TC202414; Fig. 6*A*). This protein accumulated during drought but not during the initial 72 HAR. In this case, the ^15^N incorporation rates are higher in control conditions with a trend to conform to the treatment group 96 HAR. The selection also includes a ribosomal protein (B7FMM6), only showing ^15^N incorporation during control conditions and significantly declined 2 HAR compared with C. Although ribosomal proteins are among the first responding upon drought-recovery 2 HAR (Fig. 1, Supplemental Table S2), it was neither possible to detect enhanced nor any incorporation of ^15^N leading to the conclusion that their increase in abundance was not based on external N uptake but on internal ^14^N-reuse capacity.

For further investigation of interesting putative markers, we propose several candidates based on the fact that they have been significantly accumulated during DR and showed highest discriminatory impact also on the turnover level (Fig. 6). In roots, these comprise for instance increased RIA ratios toward the end of recovery with the GroES chaperonin G7ZWL8; a ribosomal protein I3T063; the asparagine synthetase G7JZK0; a ferredoxin nitrite reductase G7JL79; porin G7K2J4 and others. In shoots, we found histone 4 (P59259) to exhibit a significantly increased RIA only within the first 24 HAR (Fig. 6*B*). It was significantly depleted upon drought stress (supplemental Table S2), suggesting an induced synthesis and/or reduced degradation in the initial phase of recovery directly reaching the control level (no significant change during recovery observed).

However, it remains difficult to explain changes in response levels (time point ratio DR/C) by turnover rates (time point to time point difference). Thus in future, it will become a challenging task to enable precise turnover rate calculations by robust normalization through the incorporation of *e.g.* internal standards. For absolute turnover rate calculations, absolute concentrations of amino acid and protein levels will obviously become necessary.

##### Concluding Remarks

This is the first study that presents *in planta* protein turnover data of plants, exposed to stress. It is also the most comprehensive analysis on drought-recovery, a feature that has not been taken much into consideration when searching for strategies to improve drought tolerance. We found evidence that drought and recovery are almost fully uncoupled processes. With combined systems biology approaches using proteomics and metabolomics data integration and correlative network analysis we gain novel insights into the plants drought-recovery plasticity. However, to what extent this reflects field conditions needs to be tested in the future. This far, it was not clear whether plants ability to recover from severe drought would depend on the outside resupply of water and thus nutrients alone or from their inherent metabolite capacity. This study revealed that a minimal capacity of amino acid as pool and/or gained through protein degradation needs to be available in the plant to enable drought-recovery before water/nutrient resupply. This capacity describes the capability of resilience and thus the maximum level of tolerance that allows for a rapid installation of the translational apparatus through *de novo* amino acid and protein synthesis prior to renutrition. An exact definition for the calculation of this capacity, however, needs to be determined in future and may vary species dependent. It remains to be found if the direct response of differential protein accumulation/synthesis after labeled N-containing water resupply in contrast to the lag-phase of ^15^N-incorporation may also involve a sensing-mechanism that induces this process. The time frame of this scenario seems to be early within the first 24 HAR. However, if this initiation cannot be established, the plants may not be able to *e.g.* induce new leaf growth because the newly available nutrients cannot be taken up in time to initiate recovery adjustment and maintenance of metabolic activity.

Previous research is aiming at identifying biomarkers responding to drought for genetic engineering and smart breeding that improve drought tolerance. However, so far little success has been achieved in Quantitative Trait Loci mapping and genome-wide association studies. This study gives evidence for a novel, thus far undetected, metabolic remobilization network that is involved in the recovery rather than stress adjustment. Hence, new strategies need to be developed to enable plants to survive drought, for instance on amino acid pool size. Crop nutritional aspects (soil nutrients as well as plants efficiency to use available nutrients) as well as field experiments should come into stronger research focus in future for improved environmental stress tolerance.

## Supplementary Material

Supplemental Data

Supplemental Data

## Footnotes

^1^ The abbreviations used are:

SILIP: Stable isotope labeling *in planta*
N: nitrogen
C: control
PS: photosynthesis
D: drought
DR: drought-recovery
TP: time point
FW: fresh weight
IS: internal standard
FDR: false discovery rate
PSII: Photosystem II
F′: chlorophyll fluorescence in the light-adapted state
Fm′: chlorophyll fluorescence when PSII centres are maximally closed in the light-adapted state
Fq′: difference between ′and Fm′
Ψxylem: xylem water potential
gs: stomatal conductance
RIA: Relative Isotope Abundance
HAR: hours after rewatering
nd: not determined.
