# Supplementary material for: Drought and Recovery: Independently Regulated Processes Highlighting the Importance of Protein Turnover Dynamics and Translational Regulation in Medicago truncatula
Source: Mol Cell Proteomics. 2016 Mar 21;15(6):1921–37. doi: 10.1074/mcp.M115.049205 (PMC5083093; doi:10.1074/mcp.M115.049205)
Supplement: Supplemental Data [file supp_15_6_1921__index.html]

Drought and recovery: independently regulated processes highlighting the importance of protein turnover dynamics and translational regulation in Medicago truncatula — Drought and Recovery: Independently Regulated Processes Highlighting the Importance of Protein Turnover Dynamics and Translational Regulation in Medicago truncatula — Molecular Regulation of Drought-Deacclimation — Supplemental Data 

# Drought and Recovery: Independently Regulated Processes Highlighting the Importance of Protein Turnover Dynamics and Translational Regulation in *Medicago truncatula*

## Supplemental Data

- Supplemental Figure 1 (.pdf, 113 KB) - Effects of water withholding and resupply on substrate water content (A) and plant physiological parameters: stomatal conductance (C), PS II operating efficiency (B) and leaf xylem water potential (D). Asterisks indicate level of significance of t‐test (\* p < 0.05; \*\* p < 0.01, \*\*\* p < 0.001)
- Supplemental Figure 2 (.pdf, 178 KB) - 2i) Go analysis of all\* shoot proteins that significantly altered in abundance during A) 10 days of drought; B) re‐watering: Biological Process \* only from protein accessions that retrieved GO annotations 2ii) Go analysis of all\* root proteins that significantly altered in abundance during A) 10 days of drought; B) re‐watering: Biological Process \* only from protein accessions that retrieved GO annotations
- Supplemental Figure 3 (.pdf, 305 KB) - 3i. Principal component (PC) analysis of the relative isotope abundances (RIAs), drought‐recovery (DR) and control (C) for shoots (A) and roots (B). Indication of a continuous increase in RIA over time (PC1) and difference of RIAs between C and DR samples (PC2). 3ii. Overview protein functional categories by the sum of absolute values retrieved from the loadings of PC 2 of the RIAs (Figure 5), indication of the impact of separation between control and drought recovery.
- Supplemental Table 1 (.pdf, 8 KB) - Information on ID specificities of the identified secondary metabolites of roots and shoots using LC-MS/MS.
- Supplemental Table 2 (.xlsx, 354 KB) - 2i. List of shoot proteins with significant changed ratios ( treatment vs. control; ANOVA Tukey p ≤ 0.05; FDR Benjamini Hochberg) 2ii. List of root proteins with significant changed ratios ( treatment vs. control; ANOVA Tukey p ≤ 0.05; FDR Benjamini Hochberg )
- Supplemental Table 3 (.xlsx, 81 KB) - 3i. PCA loadings of relative quantitative root portein and metabolite data of Supplemental Table 2ii (PCA - Supplemental Figure 2i) 3ii. PCA loadings of relative quantitative shoot portein and metabolite data of Supplemental Table 2i (PCA - Supplemental Figure 2i)
- Supplemental Table 4 (.xlsx, 6.2 MB) - 4i. Output of the automated MS-information extraction of 15N incorporation into peptides given as RIAs and the subsequent calculations of RIA ratios (DR/C) ( treatment vs. control; ANOVA Tukey p ≤ 0.05; FDR 4ii. Output of the automated MS-information extraction of 15N incorporation into peptides given as RIAs and the subsequent calculations of RIA ratios (DR/C) ( treatment vs. control; ANOVA Tukey p ≤ 0.05; FDR
- Supplemental Table 5 (.xlsx, 13.4 MB) - 5i. Detailed information on protein/peptide ID and quality assessment (Sequest and ProteomeDiscoverer) of roots 5ii. Detailed information on protein/peptide ID and quality assessment (Sequest and ProteomeDiscoverer) of shoots
- Supplemental Information Kdeg and Ksyn (.pdf, 97 KB) - Supplemental Information for Calculations of Kdeg and Ksyn
- Supplemental Information SELPEX and Turnover (.pdf, 17 KB) - Detailed description of SELPEX list generation and Protover settings.
