# Supplementary material for: Drought and Recovery: Independently Regulated Processes Highlighting the Importance of Protein Turnover Dynamics and Translational Regulation in Medicago truncatula
Source: Mol Cell Proteomics. 2016 Mar 21;15(6):1921–37. doi: 10.1074/mcp.M115.049205 (PMC5083093; doi:10.1074/mcp.M115.049205)
Supplement: Supplemental Data [file 10.1074_M115.049205_mcp.M115.049205-1.pdf]

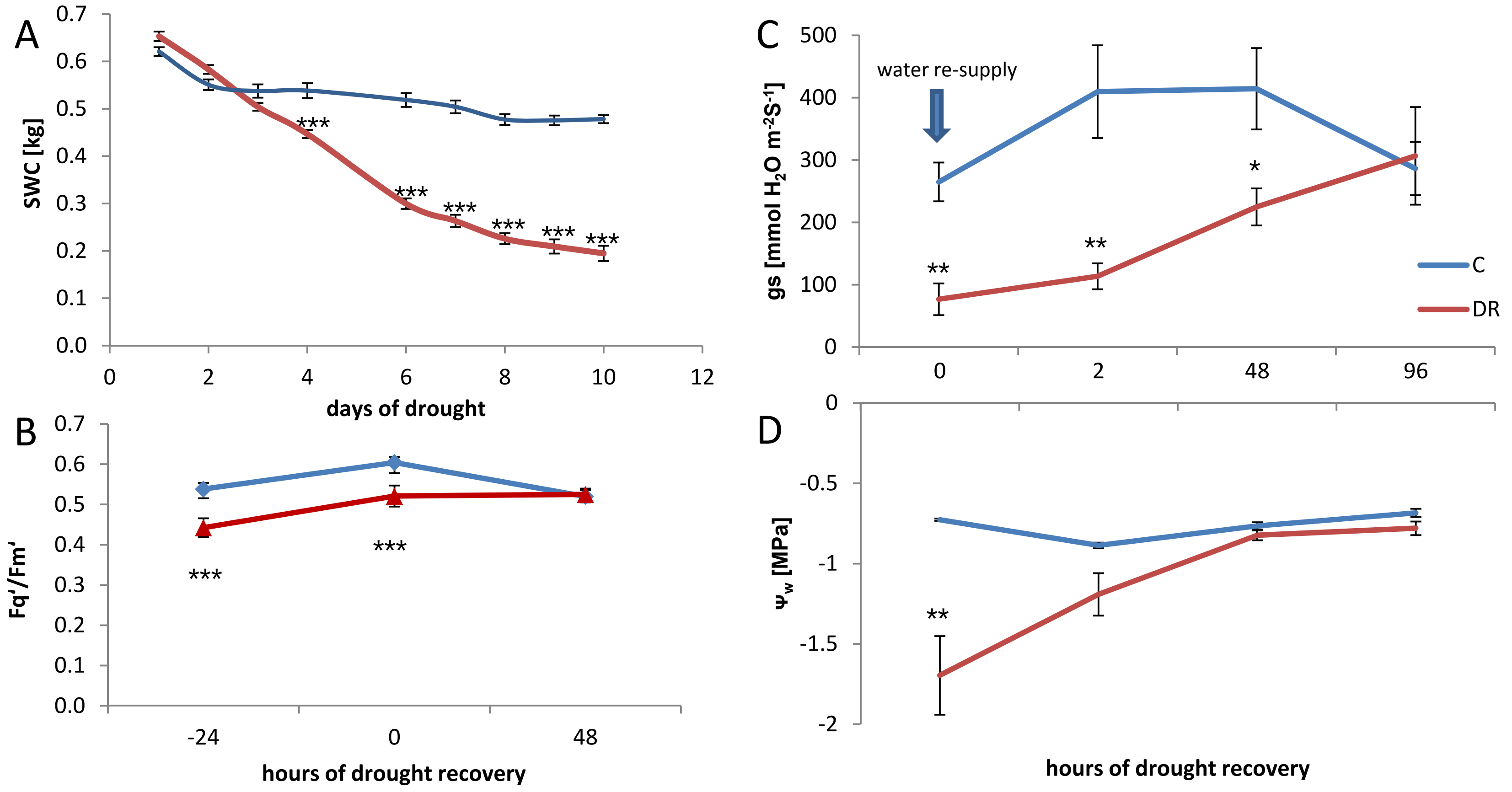


**Supplemental Figure 1.**

Effects of water withholding and resupply on substrate water content (A) and plant physiological parameters: stomatal conductance (C), PS II operating efficiency (B) and leaf xylem water potential (D).

Asterisks indicate level of significance of t-test (* $p < 0.05$; ** $p < 0.01$, *** $p < 0.001$)
