# Supplementary material for: Drought and Recovery: Independently Regulated Processes Highlighting the Importance of Protein Turnover Dynamics and Translational Regulation in Medicago truncatula
Source: Mol Cell Proteomics. 2016 Mar 21;15(6):1921–37. doi: 10.1074/mcp.M115.049205 (PMC5083093; doi:10.1074/mcp.M115.049205)
Supplement: Supplemental Data [file 10.1074_M115.049205_mcp.M115.049205-2.pdf]

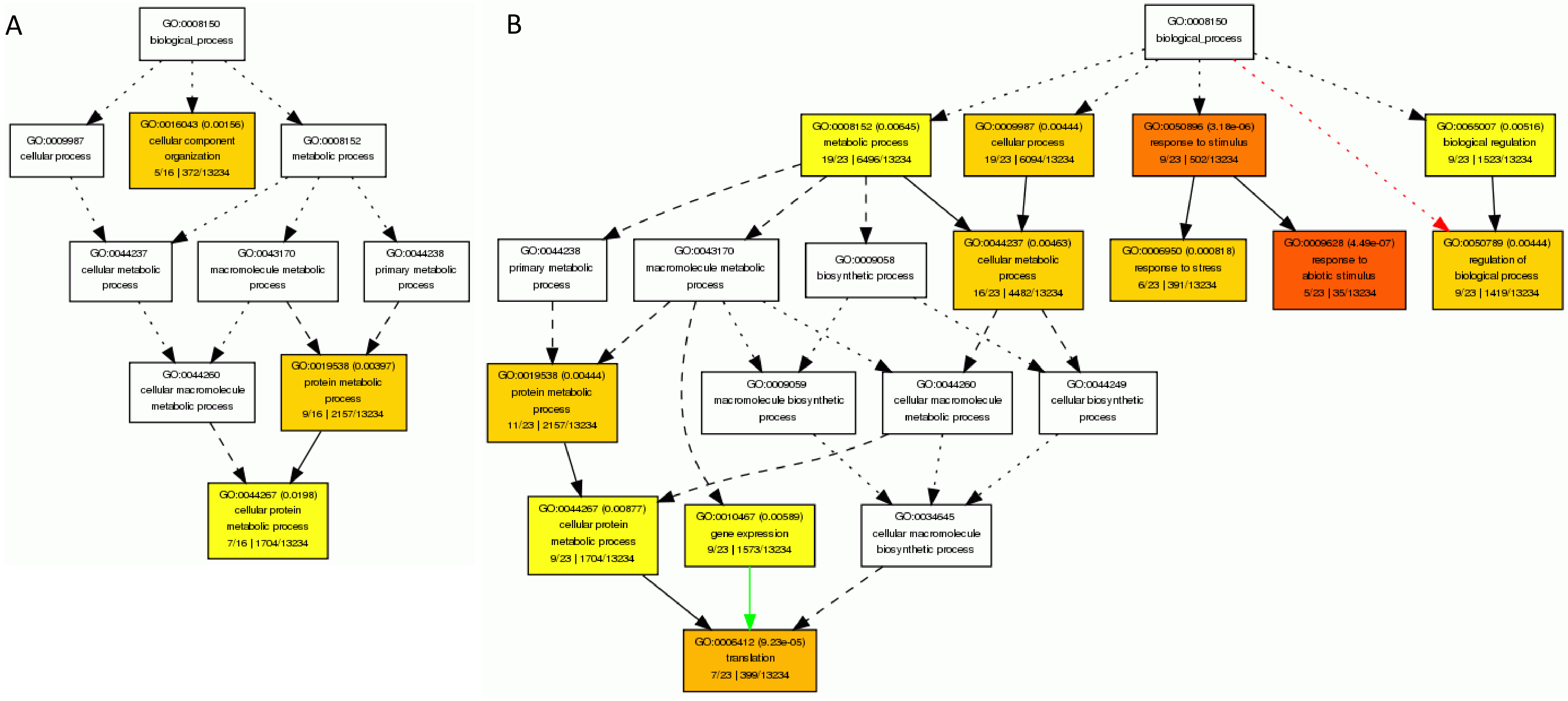

Supplemental Figure 2i)

Go analysis of all* shoot proteins that significantly altered in abundance during A) 10 days of drought; B) re-watering: Biological Process

* only from protein accessions that retrieved GO annotations

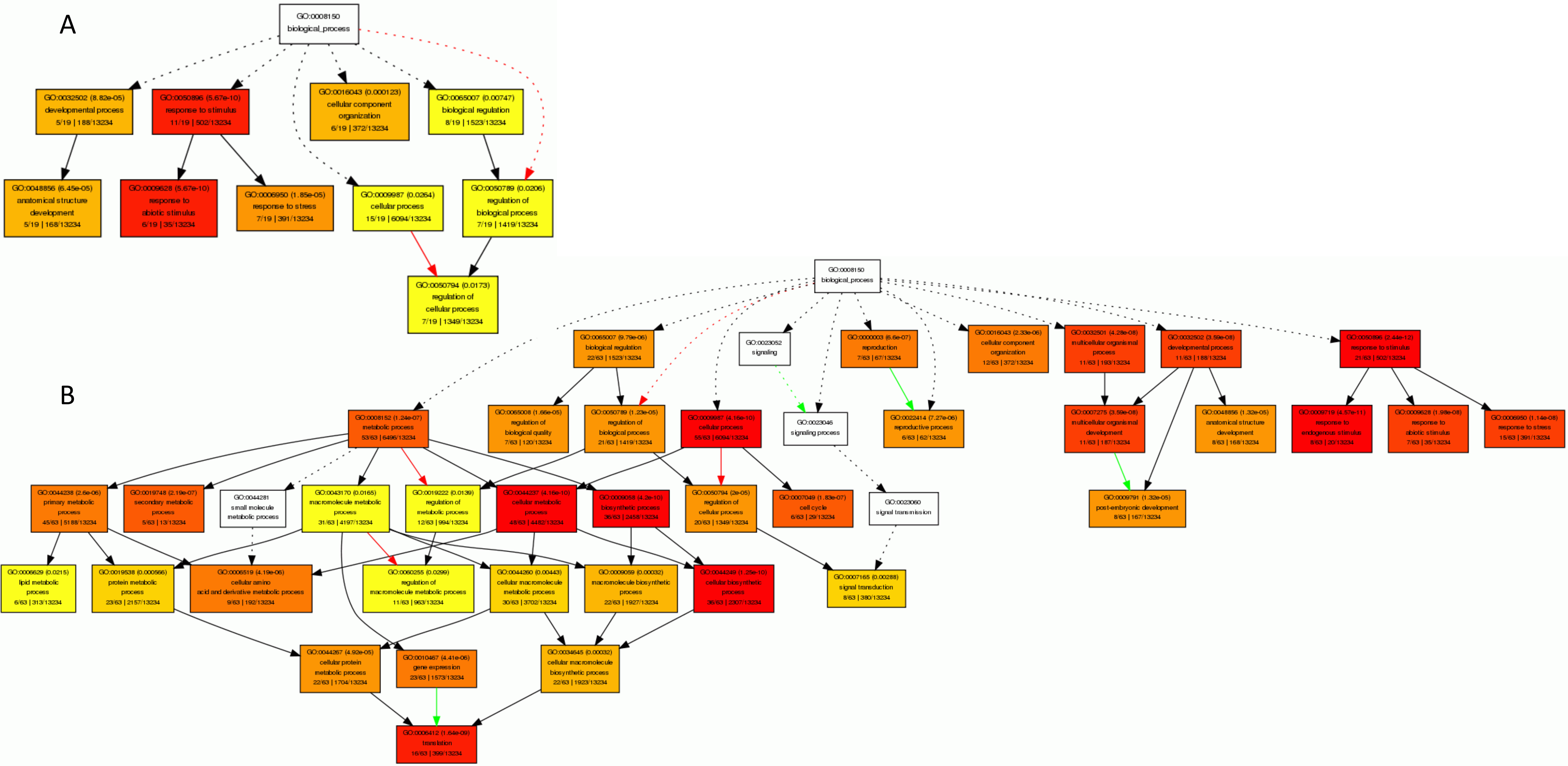

Supplemental Figure 2ii)

Go analysis of all* root proteins that significantly altered in abundance during A) 10 days of drought; B) re-watering: Biological Process

* only from protein accessions that retrieved GO annotations
