# Supplementary material for: Drought and Recovery: Independently Regulated Processes Highlighting the Importance of Protein Turnover Dynamics and Translational Regulation in Medicago truncatula
Source: Mol Cell Proteomics. 2016 Mar 21;15(6):1921–37. doi: 10.1074/mcp.M115.049205 (PMC5083093; doi:10.1074/mcp.M115.049205)
Supplement: Supplemental Data [file 10.1074_M115.049205_mcp.M115.049205-3.pdf]

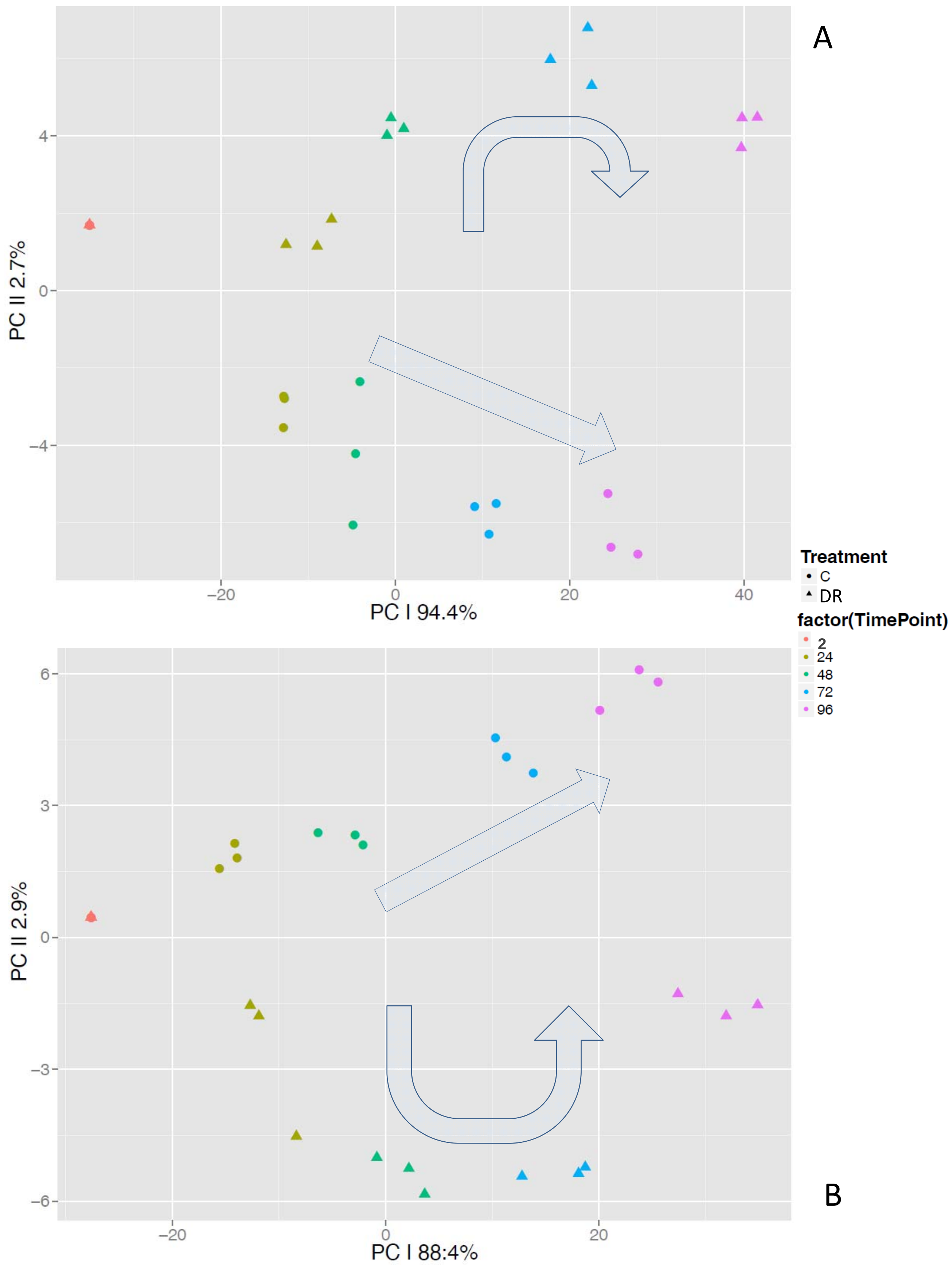

Supplemental Figure 3i. Principal component (PC) analysis of the relative isotope abundances (RIAs), drought-recovery (DR) and control (C) for shoots (A) and roots (B). Indication of a continuous increase in RIA over time (PC1) and difference of RIAs between C and DR samples (PC2).

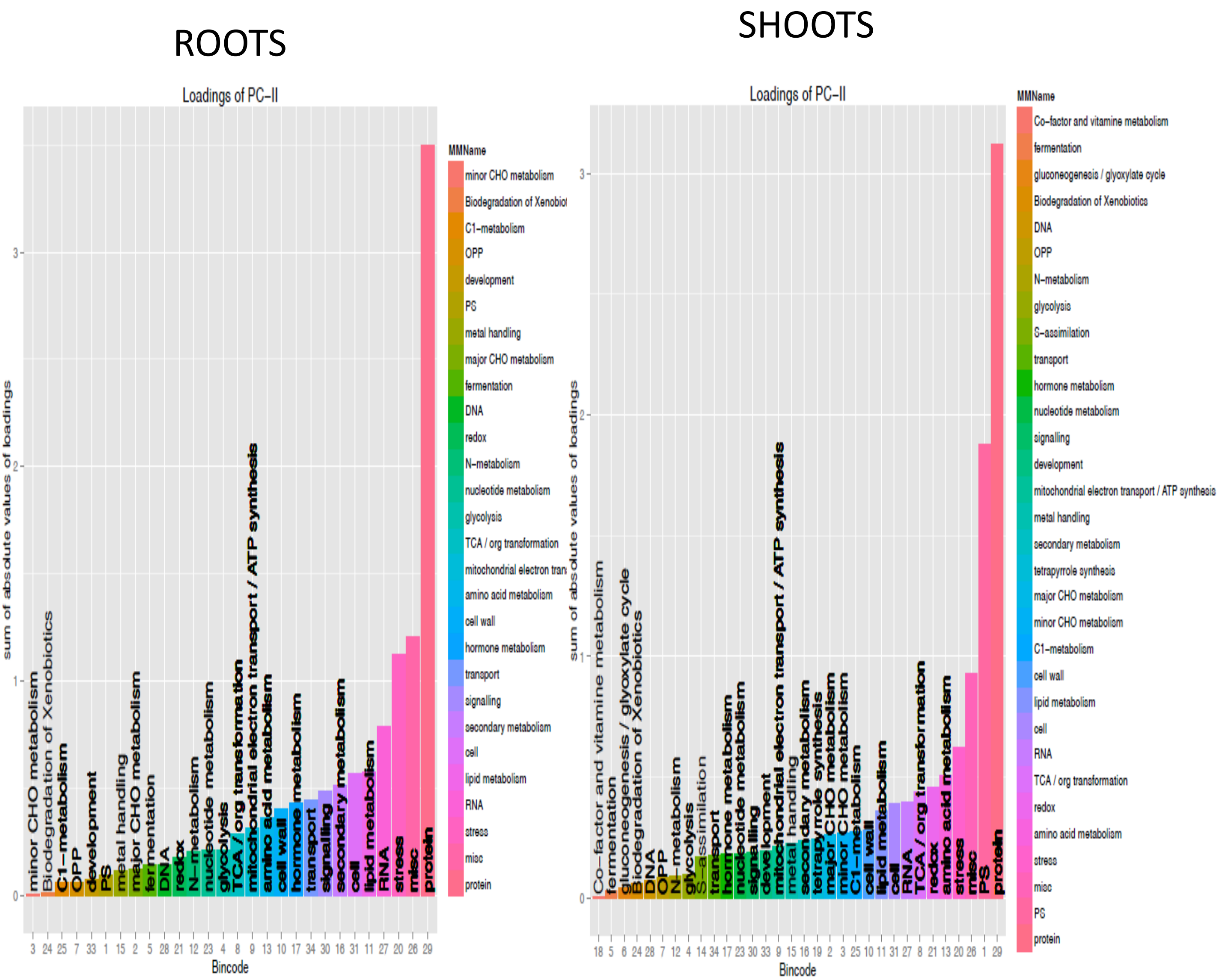

Supplemental Figure 3ii. Overview protein functional categories by the sum of absolute values retrieved from the loadings of PC 2 of the RIAs (Figure 5), indication of the impact of separation between control and drought recovery.
