# Supplementary material for: Drought and Recovery: Independently Regulated Processes Highlighting the Importance of Protein Turnover Dynamics and Translational Regulation in Medicago truncatula
Source: Mol Cell Proteomics. 2016 Mar 21;15(6):1921–37. doi: 10.1074/mcp.M115.049205 (PMC5083093; doi:10.1074/mcp.M115.049205)
Supplement: Supplemental Data [file 10.1074_M115.049205_mcp.M115.049205-4.pdf]

**Suppl Table 1.**

Information on ID specificities of the identified secondary metabolites of roots and shoots using LC-MS/MS.

| Shoots | Formula | Rt (min) | Literature | Calculated | Measured | Other peaks | Found in literature |
|---|---|---|---|---|---|---|---|
| Apigenine | C15H10O5 | 58.7 | 271.0601 | 271.0606 | 271.0601 | | Wojakovska et al. 2013 |
| Apigenine-7-beta-Glucoside | C21H20O10 | 57.4 | | 433.1134 | 433.1135 | 271 | In house standard |
| Apigenine 7-O-[2'-O-coumaroyl-glucuronopyranosyl-(1-2)-O-glucuronopyranoside | C36H32O19 | 60.4 | 769.1611 | 769.1611 | 796.1612 | 447.38; 323.22; 271.05 | Marzcak et al 2010, Kowalska et al 2007 |
| Genistein | C15H10O5 | 56.7 | 271.0601 | 271.0606 | 271.0596 | | Staszków et al. 2011, Wojakovska et al. 2013 |
| Genistein GlcA | C21H18O11 | 47.2 | 447.0922 | 447.0927 | 447.0921 | 271.06 | Staszków et al. 2011 |
| Genistein GlcA GlcA | C27H26O17 | 55.8 | 623.1243 | 623.1275 | 623.1240 | 447.09; 271.04 | Staszków et al. 2011 |
| Malvidine | C17H15O7 | 58.8 | | 331.0817 | 331.0812 | | In house standard |
| Malvidine Glc | C23H25O12 | 57.8 | | 493.1346 | 493.1341 | 331.08; 301.07 | In house standard |
| Chrysoeriol | C16H12O6 | 59.8 | 301.0707 | 301.0712 | 301.0707 | | Staszków et al. 2011, Wojakowksa et al. 2013, Stochmal et al 2001 |
| Cyanidine | C15H11O6 | 55.8 | | 287.0555 | 287.0550 | | In house standard |
| **Roots** | | | | | | | |
| Apigenine | C15H10O5 | 58.7 | 271.0601 | 271.0606 | 271.0601 | | Wojakovska et al. 2013 |
| Apigenine-7-beta-Glucoside | C21H20O10 | 59.6 | | 703.1662 | 433.1135 | 271 | In house standard |
| Genistein | C15H10O5 | 56.7 | 271.0601 | 271.0606 | 271.0596 | | Staszków et al. 2011, Wojakovska et al. 2013 |
| Genistein GlcA GlcA | C27H26O17 | 50.6 | 623.1243 | 623.1275 | 623.1240 | 447.09; 271.04 | Staszków et al. 2011 |
| Malvidine Glc | C23H25O12 | 58.7 | | 493.1346 | 493.1337 | 331.08; 301.07 | In house standard |
| Daidzein | C15H10O4 | 61.4 | 255.0649 | 255.0657 | 255.0649 | | Staszków et al. 2011 |
| Biochanin A | C16H12O5 | 58.3 | 285.0759 | 285.0762 | 285.0762 | | Staszków et al. 2011 |
