# Supplementary material for: Drought and Recovery: Independently Regulated Processes Highlighting the Importance of Protein Turnover Dynamics and Translational Regulation in Medicago truncatula
Source: Mol Cell Proteomics. 2016 Mar 21;15(6):1921–37. doi: 10.1074/mcp.M115.049205 (PMC5083093; doi:10.1074/mcp.M115.049205)
Supplement: Supplemental Data [file 10.1074_M115.049205_mcp.M115.049205-9.pdf]

Supplemental Information for Calculations of Kdeg and Ksyn:

The equations are analogous to [Martin et al. JPR 2012], however note that we have inverted the terms pairs Lm and Lm+1, tm and tm+1, as well as Hm and Hm+1, due to the fact that we measured the ^{15}N incorporation over time and not the ^{15}N loss over time.
Let Lm be the sum of peak intensities of the light isotopic envelope.
Let tm be a specific time point.
Let Hm be the sum of peak intensities of the heavy isotopic envelope.

$$K_{deg} = -\frac{L_m - L_{m+1}}{t_m - t_{m+1}} * \frac{1}{L_m}$$

$$K_{syn} = -\frac{H_m - H_{m+1}}{t_m - t_{m+1}} + K_{deg} * H_m$$
