# Supplementary material for: Drought and Recovery: Independently Regulated Processes Highlighting the Importance of Protein Turnover Dynamics and Translational Regulation in Medicago truncatula
Source: Mol Cell Proteomics. 2016 Mar 21;15(6):1921–37. doi: 10.1074/mcp.M115.049205 (PMC5083093; doi:10.1074/mcp.M115.049205)
Supplement: Supplemental Data [file 10.1074_M115.049205_mcp.M115.049205-10.pdf]

Supplemental_SELPEX and Turnover

Detailed description of SELPEX list generation and Protover settings.

For Selected Peptide Extraction list (SelPEx; 33) generation, the raw LC/MS data were processed with the ProteomeDiscoverer software as described in section 2.12. Since the automated identification of shotgun proteomics data from partial metabolic labelling experiments is not possible, we transferred identifications from the analogously treated, but unlabelled samples. Identifications from control as well as treatment sample groups, and samples of all TPs were used to generate the input for the subsequent analysis of isotopically enriched data. The thereby generated Peptide Spectrum Matches (PSMs), of all biological and technical replicates of all TimePoints (TPs) and of both treatments (3 x 2 x 5 x 2 = 60 files) were filtered according to the following criteria. A minimum of 2 peptides per protein, peptide confidence level medium or high, XCorr-score minimum of 2 and no modifications, were applied. From the remaining PSMs the amino acid sequence, the charge state and the Retention times (Rt) were used to generate the Selpex list. For each amino acid sequence, the average of the Rt (excluding missing values) was calculated, as well as the average of the charge states (excluding missing values), which was rounded down to the closest integer. This resulted in a list of non-redundant peptide amino acid sequences, their charge state and Rt, as well as their corresponding protein Accession Number. Thus, for the shoots 675 proteins with a total of 3753 peptides, and for the roots 719 proteins with a total of 3937 peptides were selected.

All raw files were converted to mzML files using the ProteoWizard msconvert-tool (Chambers et al., 2012). A RT range of 5 min and a Mass Accuracy of 10 ppm were used in conjunction with the aforementioned Selpex list as the input for the previously published software (24), generating data matrices. These contain the extracted spectral envelopes of the given peptides (m/z and intensity pairs), the sum of isotopic

peaks belonging to the light (L; natural isotopic distribution) and to the heavy (H; enriched with ^{15}N) part of the composite spectrum, as well as the Relative Isotope Abundance (RIA; (H/H+L)).

Differential protein abundance can be expected to occur, due to the treatment and the time series, and the transfer of identifications poses a potential uncertainty (since PSMs from control and treated sample groups were merged). Therefore, we cannot expect all peptide signals to be present or detectable in all time points (TPs) within both treatments. Consequentially, filters, evaluating the RIA values, were created to resolve this issue. This filtering logic was implemented using an unpublished in-house Python script.

Each of the following five filters evaluates the quality of the calculated RIA values under a different aspect, and results in the attribution of a Boolean (true or false) assignment for each peptide for each biological and technical replicate, for each TP and for each treatment. The positive result of a filter was assigned a value of 1, while a negative evaluation resulted in a 0. The sum of all five filters of all TPs of each individual peptide had to be greater or equal to three quarters of the maximum possible value, in order for the peptide to be regarded as a true positive signal.

After careful inspection of the individual and overall results of these filters, three technical replicates with reproducibility issues could be distinguished (from 24 altogether). One technical replicate within the roots and two technical replicates within the shoots data were thus removed completely from the respective data analysis.

In order to extract reproducible and biologically meaningful peptide features, initially only those peptide signals with an overall positive evaluation in all three biological replicates within only one of two treatments (C or DR) were selected. Thereby, the same peptide signals occurring either only in C or only in the DR as well as in both

sample group were selected. This procedure resulted in 673 proteins and 3603 peptides for shoots and 579 proteins and 2618 peptides for the roots. Thereby, the same peptides signals occurring in both or only in the control or only in the treated sample groups were selected and used for comparison. This, however, also means that when comparing the signals for a given protein one of the treatments might have e.g. 5 peptides for the protein within C but only 3 peptides for the same protein in DR. These data where thus only used to extract proteins that exclusively showed ^{15}N incorporation in one of the treatments.

For all other analyses, the data where further filtered such that peptide signals with an overall positive evaluation in all three biological replicates as well as their technical replicates and occurring within both given treatments were selected. This procedure resulted in 657 proteins and 3076 peptides for shoots and 428 proteins and 1448 peptides for the roots (Supplemental Table 5).

Filter #1: RIA increasing with time (evaluates interdependent LC/MS measurements of 5 time points (time series) of a single biological and technical replicate):

This filter evaluates the trend of the peptide RIA value within 5 interdependent LC/MS measurements (time series) of a single biological replicate. In chronological order (from natural abundance to ^{15}N isotopic enrichment) the RIA of an individual peptide has to either increase or the absolute value of the difference between RIA_{TPn} and RIA_{TPn+1}, has to be smaller than a fourth of RIA_{TPn+1}. Therefore, RIA values have to increase with time, but fluctuations up to a maximum of 25% are allowed.

Filter #2: RIA reproducibility of peptide (evaluates RIA reproducibility of a given time point between biological and technical replicates)

This filter evaluates the reproducibility of all technical and biological replicates of the RIA values of a single peptide of a single time point. Separately calculate the mean and standard deviation of the peptide RIAs of all biological and technical replicates,

for each treatment and for each time point. Individual RIA values within +/- 2 times the standard deviation passed the filter, while the others did not.

Filter #3: Coverage reproducibility of peptide (evaluates RIA reproducibility of a given time point between biological and technical replicates)

Analogous to filter #2, this filter evaluates the reproducibility of all technical and biological replicates of the coverage values (number of picked peaks divided by the number of theoretically possible peaks, see (24)) of a single peptide. It separately calculates the mean and standard deviation of the peptide coverage of all biological and technical replicates, for each treatment and for each time point. Individual RIA values within +/- 2 times the standard deviation passed the filter, while the others did not.

Filter #4: Outlier detection of protein RIA (evaluates the presence of outliers by comparing RIA values of all peptides associated with a given protein accession at individual time points within a given treatment).This filter tries to find outliers of individual peptide RIA values of a specific replicate, by comparing it to the entirety of peptide RIA values of the associated protein. Separately calculate the Inter Quartile Range (IQR) of the RIA of all peptides associated with a given protein for each treatment and for each TP. RIA values of individual peptides within 1.5 times the IQR passed the filter, while others did not.

Filter #5: RIA missing values (evaluates interdependent LC/MS measurements of 5 time points (time series) of a single biological and technical replicate)

No more than half of the RIA values may be missing (i.e. a maximum of 2 out of 5 time points).

The mean RIA value of a peptide for each individual time point was calculated by averaging over both technical replicates, resulting in an averaged RIA value for each

peptide for each time point for each biological replicate and for each treatment. These peptide RIA values were again averaged to get protein RIA values.

This procedure resulted in 657 proteins and 3076 peptides for shoots and 428 proteins and 1448 peptides for the roots (Supplemental Table 3 and PRIDE repository dataset PXD 001728).

This data were used for the following statistical visualizations using the “prcomp” and “ggplot2” library in R: PCA of RIAs, Hierarchical cluster analysis (Eucledian distance) of RIA ratios (DR/C) and BoxPlots of putative marker proteins.

Additional Citation

Chambers, M. C., Maclean, B., Burke, R., Amodei, D., Ruderman, D. L., Neumann, S., … Mallick, P. (2012). A cross-platform toolkit for mass spectrometry and proteomics. *Nat Biotech*, *30*(10), 918–920.
