# Supplementary material for: Drought and Recovery: Independently Regulated Processes Highlighting the Importance of Protein Turnover Dynamics and Translational Regulation in Medicago truncatula
Source: Mol Cell Proteomics. 2016 Mar 21;15(6):1921–37. doi: 10.1074/mcp.M115.049205 (PMC5083093; doi:10.1074/mcp.M115.049205)
Supplement: Supplemental Data [file supp_15_6_1921_v2_index.html]

Drought and Recovery: Independently Regulated Processes Highlighting the Importance of Protein Turnover Dynamics and Translational Regulation in Medicago truncatula — Molecular Regulation of Drought-Deacclimation — Supplemental Data 

# Drought and Recovery: Independently Regulated Processes Highlighting the Importance of Protein Turnover Dynamics and Translational Regulation in *Medicago truncatula*

## Supplemental Data

**Files in this Data Supplement:**

- Supplemental Table
